# Work intensification: a theoretical and practical issue for nursing practice

**DOI:** 10.1590/1980-220X-REEUSP-2025-0408en

**Published:** 2026-05-01

**Authors:** Helena Maria Scherlowski Leal David, Hugo Pinto de Almeida

**Affiliations:** 1 Universidade do Estado do Rio de Janeiro, Faculdade de Enfermagem, Programa de Pós-Graduação em Enfermagem, Rio de Janeiro, RJ, Brazil.; 2 Universidade do Estado do Rio de Janeiro, Faculdade de Enfermagem, Rio de Janeiro, RJ, Brazil.

**Keywords:** Nursing, Healthcare Work Process, Workload, Working Conditions, Occupational Health

## Abstract

**Objective:**

Discussing from a theoretical-practical perspective, the phenomenon of work intensification and its implications for nursing practice and workers’ health.

**Method:**

Based on a literature review, through a non-systematic search on work intensification in nursing, combined with the authors’ experience and critical thinking.

**Results:**

Grounded on the critical theory of labor value, this study analyzes the characteristics of nursing work and the influences of work organization forms in relation to current modes of accumulation. It discusses how intensification manifests itself in different scenarios within the health sector and its repercussions on work and workers’ health.

**Final considerations:**

Although it is not yet a widely established research topic, work intensification appears in various forms in the academic literature. To advance our understanding of the subject, it is essential to consider recent changes in the organization of work in health services. In a context of struggles for better working conditions and reduced working hours, it is crucial that associations and unions address this issue.

## INTRODUCTION

The nursing profession has lately revealed an interest in one of the core aspects of its practice—the heavy workload, excessive demands, burnout, and risks imposed upon workers. Unlike industrial, agricultural, or commercial production processes, in which raw materials are transformed, healthcare work has human life as its direct object. In this context, its practice involves dimensions that go beyond strictly technical competencies, including the ability to deal with the suffering of the people who are the objects of care^(1)^.

Healthcare professionals often find it difficult to recognize the conditions that may contribute to the occurrence of health issues, illnesses, and accidents within their own work process. It can be frequently observed a strong commitment to care, in which, even in the face of inadequate conditions, workers direct their energy toward attending the individual and the community. In this context, critical reflection and questioning of their own working conditions tend to play a secondary role, leaving them subjected to various forms of physical, emotional, and cognitive overload^(2)^. However, when discussing workloads or occupational risks, one must ultimately consider the development of irreversible and disabling pathological conditions, many of which could be prevented. Furthermore, health interventions targeting the determinants and factors underlying occupational health problems should aim, in a broader sense, to promote workers’ health from a enlarged perspective of quality of life, rather than merely focusing on the isolated prevention of specific problems.

It is worth briefly discussing the distinction between the concepts of occupational risk and workloads. The concept of occupational risks has been widely used within the theoretical framework of traditional epidemiological studies, anchored in the perspective of occupational health and occupational hygiene—approaches that do not delve into analyses of the historical and political roots of workers’ health.

In turn, the concept of workload, formulated and developed primarily within the context of Latin American Social Medicine, stems from an understanding of health grounded in the perspective of its social production—that is, seeking to understand health phenomena as historical and produced under specific social conditions and relationships. The notion of occupational risks, despite being useful for identifying isolated elements of work capable of increasing the likelihood of health damage, is insufficient because it fails to account for the complexity of the wear-and-tear process, attributing specific diseases to specific agents and ignoring the broader pathogenesis. It is also ideological because, by focusing on external agents, it shifts the focus away from the social organization of capitalist labor—which is the true root of wear and tear—toward technical and palliative solutions that do not challenge the exploitative logic of capital. Therefore, while “occupational risks” is a category that individualizes, fragments, and technifies the problem, “workload” is a category that socializes and politicizes the issue, placing the struggle for the transformation of working conditions and the organization of labor at the center of the debate^(3)^.

Changes as a result of the process of productive restructuring have brought about modifications in the nature, content, and meaning of work^(4)^. In recent decades, processes of technological modernization and the reorganization of productive structures have produced significant changes in labor management. Among the main characteristics of this trend are the intensification of the work pace, the extension of working hours, the standardization and mechanization of tasks, stricter control of activities, the incorporation of targets into evaluation processes, pressure exerted by management, the demand for versatile workers, and the precariousness of labor relations, among other aspects^(4)^.

Starting in the 1990s, a series of new issues also emerged in the field of healthcare labor aspects, marking a turning point in the analysis and research on the healthcare work process. On the one hand, concerns arose regarding new forms of flexible or informal work, as well as state-promoted regulation, which focused on the institutional mechanisms that manage work^(2,5)^. On the other hand, there are issues regarding the comprehensiveness of care and the autonomy of individuals, with the focus of analysis shifting to the interactions involved in the relationship between professionals and users, or among the professionals themselves^(6)^.

The distinction between productive labor, as surplus value producer, and unproductive labor, as a realizer of surplus value—which is particularly relevant in the analysis of capital itself—does not present itself with the same clarity in the realm of concrete relations. As indicated by various Marxist interpretations^(7)^, being a productive or unproductive worker immediately implies being exploited by capital. Within the strict scope of capitalist production^(8)^, the difference does not lie in what is produced—a material good or a service—but for whom and under what social relations the work is performed. On the theoretical level (the “capital in itself”), the distinction is clear and essential for understanding the genesis of surplus value, which is the secret of capitalist accumulation: productive labor is the source of capitalist wealth. On the level of concrete relations (the “phenomenon appearance”), however, this distinction fades away. Unproductive labor is functional and indispensable. The wage form unifies disparate experiences, and the increasing complexity of capitalism (especially in dependent countries like Brazil) creates a *continuum* of exploitation, in which the line separating productive from unproductive labor is crossed by relations of precariousness, the real subsumption of labor under financial capital, and an immense mass of workers who, without producing surplus value, are vital to its realization and to the reproduction of cheap labor^(8,9)^.

The nursing workforce, like that of other categories comprising the working class, is subject to recent transformations in the management and organization of work^(2)^. In this context, the fragmentation and reorganization of nursing activities, often justified by the pursuit of greater efficiency and productivity, tend to treat this workforce as a commodity to be mobilized according to the demands of the production process^(1)^.

The challenge, therefore, is to identify and analyze the mediations between the macrostructural dimensions of the world of work and wealth production, and the everyday spheres of healthcare work—mediations that affect and shape specific modes of work organization. From this perspective, it is also worth critically pointing out the challenges posed to occupational health approaches, in contrast to the limitations of the fields of occupational health and occupational medicine^(10)^. The literature records a significant number of studies dedicated to the health of nursing workers. Much of this research, however, focuses on describing the diseases and occupational risks to which these professionals are exposed^(11)^. Although relevant, such approaches tend to offer a partial understanding of reality, since they often fail to examine the determinants of work-related morbidities, limiting themselves to isolated interventions and failing to analyze the structural origins of these problems.

The health of nursing professionals is influenced by various dimensions of work organization, notably working hours, remuneration, degree of autonomy, working conditions, and socio-professional relationships^(12,13)^—factors that have also been identified as associated with nurses leaving the profession in European countries^(14)^. In this sense, by employing the concept of work intensification as the object of analysis, this study delves deeper into issues identified in previous studies, in which changes and configurations in the organization of nursing work were already presented as an important determinant of health-disease processes among workers in this field, beyond a narrow focus on specific risks to which they might be subjected.

This study is an academic production by a certified research group focused on the relationships between work, workers’ health, and nursing, linked to a graduate program in the field of nursing, comprising researchers at different levels of training. The objectives of this study are to characterize and describe, from a theoretical-conceptual perspective, the organization of nursing work within the capitalist mode of production and to discuss the phenomenon of work intensification and its implications for nursing work and worker health.

## METHOD

This is a theoretical-reflective study, grounded in contemporary scientific literature on the topic of work intensification in nursing and anchored in a critical analytical perspective. In the first phase, between 2020 and 2025, a non-systematic search was conducted for studies directly addressing the subject of study. The search filter combined “*work intensification AND nursing*,” using the following databases: Scientific Electronic Library Online – SciELO, Latin American and Caribbean Health Sciences Literature – Lilacs, and the Brazilian Digital Library of Theses and Dissertations (BDTB). In the SciELO database, four texts were selected, two of which were considered relevant to the topic. In the Lilacs database, no articles were found. In the BDTB database, 31 theses or dissertations were found, and two were selected that addressed work intensification as a central theme. After filtering out duplicates, three studies were selected. The literature review was enriched by a series of articles that did not directly address the study’s main focus. However, during the course of the research, elements related to the intensification of nursing work emerged.

After comparing the available literature with the body of discussions developed in study groups and educational settings in the nursing field, the study was organized around two thematic axes: theoretical-conceptual dimensions of nursing work and its organization within the capitalist mode of production; and work intensification and its relationship to nursing and workers’ health. Based on the critical theory of labor value, the main points of discussion were the characteristics of nursing work and its organization in light of current modes of accumulation; and how the intensification of nursing work manifests in various scenarios within the healthcare sector, along with its consequences for workers’ labor and health.

## RESULTS

### Theoretical and Conceptual Dimensions of Nursing Work and its Organization within the Capitalist Mode of Production

Research on nursing work gained momentum starting in the 1980s, being approached from various theoretical perspectives. From the perspective of work as a social practice, some authors consider nursing work to be conditioned by the hegemonic biomedical care model, that is, linked to and subsumed under medical work^(15)^. On the other hand, there are also those who analyze nursing work as part of the broader field of health care, in which different professionals collaborate to develop specific processes for understanding and intervening in the health and disease processes of both individuals and the community^(6)^.

To conceptualize the health work process, we based our analysis on theoretical formulations that understand it through four interrelated dimensions: the activity itself (what is being done), the object of work, the agents (workers), and the instruments or means of work^(6,16)^. The way these elements are defined, linked, and articulated temporally and spatially expresses a specific form of work organization. With the development of capitalism, the praxis of health comes to constitute the relationship between the labor process and the process of valorization, thus being subordinated to the continuity of the process of capitalist accumulation and the reproduction of economic, political, and ideological conditions. If, on the one hand, the development of this dynamic results in the formation of a medical-industrial/financial complex that profits from the buying and selling of *health* as a commodity; on the other hand, it is also sustained by the exploitation of the workforce responsible for the production of healthcare^(17)^.

Based on classical critical analyses, particularly what has been termed the *labor theory of value* in Marx, it is considered that the organization of labor, under the capitalist mode, ceases to belong to the worker and progressively passes into the hands of those who hold the means of production^(8,18)^. From the perspective of Marxian critique, productive and unproductive labor are not separate when considering the process of capital accumulation. This may explain, in part, how different forms of management, organization, and division of labor—such as productive and unproductive labor—ultimately rest on the same theoretical-political, scientific, and pedagogical assumptions.

It is the subsumption of labor under capital that leads to the establishment of certain forms of management and administration of the labor process. In this context, the formulations within the framework of Scientific Management^(19)^ were pioneering and constituted a set of principles that came to regulate workers’ lives, both inside and outside the workplace, with the aim of increasing the exploitation of labor and reducing production costs; this logic has also been used throughout history to drive other, more flexible models of work organization, such as the Toyotist and Tensioned flow (*flux tendu*)^(2)^ models.

For the proposed reflection, a detailed exposition of the peculiarities of the different systems of work organization is not necessary, but rather an understanding that, in some way, these modes of organization have influenced and continue to influence collective work in healthcare and, specifically, in nursing^(1,2)^. From this perspective, there is no universal model for nursing work that can be applied to any healthcare institution or unit. This is because the variety of nursing activities, which accompanies the diversification of service offerings, units, and sectors, implies the use of various models aimed at reducing operational costs and increasing professional efficiency. While nursing practice exhibits distinct characteristics, management also faces forms of resistance—both individual and collective—from workers who challenge the changes implemented in their work environments^(5)^.

In this sense, nursing work shares with other categories of healthcare workers the experience of conditions and changes in the forms of work organization imposed on them by current models of accumulation. Some of these transformations can be summarized as follows: the precariousness and flexibilization of working and employment conditions (with a certain emphasis on issues of outsourcing); changes in workers’ professional qualifications and training, with an emphasis on the primacy of an instrumental *pedagogical rationality* over a formative one; and the implementation of programs derived from the so-called *Japanese model*, such as total quality management, *just-in-time*, etc., particularly in the private health sector.

Transformations in the public sector, however, became more pronounced starting in the 1990s, when a new role for the state was established. In this context, the third sector emerged as a response to the claim that public administration was incapable of using financial resources efficiently. This led to a gradual replacement of the hierarchical public bureaucracy in the provision of services to the population with the principles of business management focused on measuring results, increasing managerial autonomy and the efficiency and effectiveness of services through performance and competition^(2,20)^. Thus, the evolution of capitalism has resulted in significant transformations in the organization of nursing work, increasing job insecurity and demanding greater physical, psychological, and intellectual effort from workers. In this context, the phenomenon of work intensification has become a central aspect of analysis in the current transformations of production processes^(4,21)^.

### Work Intensification and its Relationship to Nursing and Workers’ Health

Despite the growing relevance of the debate surrounding work intensification, this is not a recent phenomenon and was already noted by Marx as present in the early days of capitalism^(4,21)^. Work intensification can be described as the denser filling of the gaps in working time with the aim of squeezing a greater *quantum* of work into the same time interval, namely the workday. This phenomenon is described in classic studies of the scientific organization of labor^(19)^, which note that increased productivity was accompanied by a reduction in the workday and a decrease in the workforce among the employees responsible for inspecting the quality of steel balls intended for the manufacture of bicycle bearings. Previously, 120 female workers worked approximately 10.5 hours per day; after analyzing the work process, only 35 worked 8.5 hours per day, managing to perform their tasks with greater precision, at a speed 2/3 faster.

Among the changes implemented to increase productivity and reduce working hours, the following stand out: the careful selection of more qualified workers, daily documentation of the quality and volume of work performed by the workers, piece-rate pay along with an additional bonus, and the addition of a 10-minute break after 1.5 hours of work. There is, therefore, a clear relationship between the concept of intensification and that of working time. Historically, the reduction of the workday has been associated with the idea of social progress and the improvement of living conditions, constituting an important agenda item in workers’ collective struggles in different national contexts^(22)^.

Two important milestones in Brazil related to the length of the workday stand out: the regulations enacted in the 1930s, later incorporated into the Consolidation of Labor Laws, and the developments stemming from the metalworkers’ strikes in the ABC region of São Paulo in the 1980s, which contributed to the reduction of the weekly workweek from 48 to 44 hours in the 1988 Federal Constitution^(22)^. Nevertheless, following these more recent achievements, new forms of work intensification began to emerge. It is important to note that this issue is particularly relevant to nursing in Brazil today, given the approval of the minimum wage for the profession (Law No. 14,434, of August 4, 2022) and the consideration in the National Congress of several other proposals (Bill No. 2,295/2000 and Proposed Amendment to the Constitution No. 19/2024) aimed at establishing a maximum workweek of 30 hours for all nursing professionals.

Without denying the importance of the achievement of reduced working hours, which will certainly contribute greatly to the professionalization of nursing work, it is necessary to question whether, on the other hand, this reduction might not lead to an intensification of work—a phenomenon observed in other professions. As mentioned earlier, in the management of health services, economic and institutional assumptions interact, shaping and defining the logic of work organization to accumulate or save capital^(20)^. This means that, even with a reduction in working hours, these assumptions remain and may give rise to practices (whether existing or new) that intensify the work of nursing professionals.

The literature has pointed to work intensification as a particular dimension of the process of exploitation and expropriation of workers’ knowledge, and it has occurred through a specific combination of various elements of the work process, such as the extension of working hours, the increase in work pace, demands for versatility, performance-based management, or stress-based administration^(21)^. Despite some studies^(13,23,24)^, there is a lack of research in which the intensification of work in nursing and healthcare appears as a specific research focus. On the other hand, a series of analyses highlight the phenomenon of intensification by pointing out the presence of elements that are closely related to or involved in the dynamics of nursing work in healthcare services and units. The literature describes how extended working hours have been implemented through the time bank system, extra shifts, and the inability of workers to actually take a vacation.

The time bank system has been adopted in both public and private hospitals due to staff shortages and management guidelines^(25, 26)^, which contributes to increased stress among nursing staff, as institutional requirements prevent them from taking time off to compensate for overtime worked when they wish to do so^(26)^. As Felli^(25)^ points out, it is common for employees to be pressured by management to make up overtime during periods of leave due to health issues, even when they would be entitled to time off due to an accident or a doctor’s note.

Extra shifts, as a form of extending the workday, are a recurring practice in the profession. This dynamic stems both from the insufficient number of professionals in healthcare units and services and from the low pay of the workforce, which does not guarantee their own and their families’ livelihood. This practice was described among workers in the sector assigned to a teaching hospital in the state of Paraná^(27)^ and to an oncology treatment unit at a specialized hospital in the state of Rio de Janeiro, where an average of 50 weekly work hours was recorded, performed to address the shortage of staff and supplement income in light of low wages^(28)^.

The final aspect of extended working hours described in studies is the failure to take vacation time during the scheduled period. According to data from a 2014 study on the profile of nursing in Brazil, it is estimated that approximately 200,000 nursing professionals, representing around 11% of the total workforce, reported not taking annual leave on a regular basis^(12)^. Since then, this number may have grown even further due to increased outsourcing and new forms of employment resulting from changes in labor legislation.

By reducing intra- and inter-shift rest breaks, the workforce’s recovery time is diminished, as the days that should be used for study, leisure, or—by right—to share in social life with family and friends, are incorporated into the weekly or monthly work schedule. Long working hours increase physical and emotional exhaustion and can lead to a range of health issues, such as chronic noncommunicable diseases^(27)^ and impaired sleep patterns^(28)^.

The second element contributing to the intensification of work lies in the increased work pace, which results from the reduction or elimination of intra- and inter-shift breaks (*pores*) during the workday. Given the diversity of services and units, the increased pace of nursing work involves factors such as a reduction in staffing levels for providing necessary care^(2,26)^, a high volume of demands to be met within a given timeframe^(2,5)^, the incorporation of technology^(13)^, or the establishment of performance evaluation targets^(5)^.

The high work pace was described as a factor intensifying nursing work in medical and surgical units at the University Hospital of Santa Maria^(23)^. Added to this process are high patient demand, a reduced number of professionals, the accumulation of multiple tasks throughout the workday, and inadequate working conditions. Martins^(5)^, in studying the restructuring of primary care in Rio de Janeiro, describes the intensification of nursing work as a result of the increased number of patient visits throughout the day and the treatment of nursing work as an extension of medical work.

Setting of targets as a form of continuous worker evaluation is used as a management tool to intensify work across various production processes^(4,20)^, an element not considered in the instrument for measuring work intensification^(24)^. However, this practice has become increasingly prevalent in nursing work. An example can be observed in the relationship between the State and social organizations (SOs), in which the management contract establishes a series of objectives and targets^(20)^ that condition the organization of work in health units. In this context, the immediate purpose of the service production process tends to take the form of a quantifiable product, expressed in the number of procedures, interventions, consultations, and other healthcare acts performed^(6)^. In work processes, targets are not merely a numerical benchmark, as they come to define the length and intensity of the workday, as well as lead to reduced breaks on the part of the worker themselves. Furthermore, they can influence actual and potential wages and generate greater control and competition among workers, while simultaneously reinforcing heteronomy, creating an environment conducive to the emergence of various forms of violence, in accordance with the established hierarchy^(4)^.

This rationality centered on quantifiable goals and results resonates with another contemporary dimension of work: the growing incorporation of digital technologies. The introduction of digital and automated technologies—linked to the so-called Industry 4.0 paradigm—has brought about profound transformations in the organization and work experience of workers across a wide range of economic sectors^(29,30)^.

Despite the scarcity of studies on this topic in nursing, it is essential to consider the potential impacts and mediations produced by tools such as electronic medical record systems, hospital management software, and new forms of platformization in healthcare work, which can reshape work schedules, modes, and relationships.

Such innovations, although legitimized by the discourse of efficiency and modernization, may be contributing to intensifying work rhythms and expanding control, reinforcing the trend toward measurement and surveillance that already characterizes the daily routine of nursing work^(4)^, as described in the platformization of work among different workers in the service sector^(30)^ or in medical work^(31)^.

Platformization, in this sense, represents an advanced stage of this process, in which technological mediation reconfigures forms of subordination, labor relations, and the very dynamics of service provision, introducing new dimensions of precariousness and burnout into the daily lives of professionals^(30,31)^. This is a clearly political phenomenon of reorganizing exploitation, in which the intensification of labor takes on new forms—whether through spatial displacement and control mediated by digital communication tools, or through the constant on-call status of these workers via connected devices^(30)^. This intensification is masked by the appearance of autonomy and pseudo-freedom of the worker, which takes on attractive contours in contexts that aim to promote the figure of the “entrepreneur.” The real-world shows, however, that this is an exploited, exhausted, precarious worker, without social protection and subjected to indefinite working hours, whose autonomy is merely an ideological means of masking subordination.

## FINAL CONSIDERATIONS

In conclusion, it follows from this discussion that, through an analysis of the concept of intensification, it is possible to examine the relationships between levels of determination and conditioning of nurses’ health in order to shed light on less visible aspects related to recent processes of change in work organization. This brings to light a new challenge: the need to adapt the theoretical and methodological approach, as well as the methods and tools, to such changes, in order to advance the analysis of the intensification of nursing work as a research subject. To this end, it is necessary not only to understand the dynamics of health services—which structure and establish the form of work organization—but also the specific characteristics derived from the experiences and lived realities of the workers themselves, which tend to obscure forms of labor exploitation sustained by processes of subjectivation. It should be noted, however, that given the complexity of the phenomenon of intensification and its determining processes, it is necessary to indicate the limits of this text, which sought to address only some of the more visible and well-documented elements in the literature, as well as recent debates. Therefore, methodological and theoretical categories of Marxian thought—particularly those of mediation and totality—were not explored, as this would require the development of a longer and more dense body of material, incompatible with the format of a reflective article.

In addition to understanding the assumptions present in health services, three aspects stand out for the study of work intensification in nursing: first, the need to understand nursing work within the context of collective labor in health, since these elements can manifest themselves in various ways across different categories of the health sector, affecting the collective of workers; secondly, the identification of forms of resistance—both overt and covert—that challenge manage­ment’s role regarding the practices of work intensification used as a mechanism to weaken workers’ collective capacity to defend their own health. Thirdly, it is essential to understand that, beyond being an academic subject to be explored, work intensification manifests itself in workers’ daily lives, therefore creating the need for a debate within professional associations and unions.

Finally, it is worth highlighting that, against the backdrop of struggles for more adequate working conditions and shorter work hours, it is essential to address work intensification as an issue that, whether tangibly or potentially, poses a risk to the quality of life and working conditions of nursing staff.

## Data Availability

The entire dataset supporting the results of this study was published in the article itself.
