# Supplementary material for: Work intensification: a theoretical and practical issue for nursing practice
Source: Rev Esc Enferm USP. 2026 May 1;60:e20250408. doi: 10.1590/1980-220X-REEUSP-2025-0408en (PMC13159548; doi:10.1590/1980-220X-REEUSP-2025-0408en)
Supplement: Translation

# Translation: A intensificação do trabalho: uma questão teórico-prática para o trabalho da enfermagem

**DOI:** 10.1590/1980-220X-REEUSP-2025-0408pt

**Authors:** Helena Maria Scherlowski Leal David, Hugo Pinto de Almeida

**Affiliations:** 1 Universidade do Estado do Rio de Janeiro, Faculdade de Enfermagem, Programa de Pós-Graduação em Enfermagem, Rio de Janeiro, RJ, Brasil.; 2 Universidade do Estado do Rio de Janeiro, Faculdade de Enfermagem, Rio de Janeiro, RJ, Brasil.

**Keywords:** Enfermagem, Processo de Trabalho em Saúde, Cargas de Trabalho, Condições de trabalho, Saúde do Trabalhador

## Abstract

**Objetivo:**

Discutir, em perspectiva teórico-prática, o fenômeno da intensificação do trabalho e suas implicações para o trabalho de enfermagem e a saúde dos trabalhadores.

**Método:**

Fundamenta-se na produção bibliográfica, por meio de busca não sistemática sobre intensificação do trabalho na enfermagem, associada à experiência e ao pensamento crítico dos autores.

**Resultados:**

Com base na teoria crítica do valor-trabalho, analisam-se as características do trabalho em enfermagem e as influências das formas de organização do trabalho frente aos modos de acumulação em curso. Discute-se como a intensificação se manifesta em diferentes cenários do setor saúde e suas repercussões no trabalho e na saúde dos trabalhadores.

**Considerações finais:**

Embora não se configure como tema de pesquisa amplamente consolidado, a intensificação do trabalho surge de distintas formas na literatura acadêmica. Para avançar na compreensão do objeto, torna-se fundamental considerar as recentes transformações na organização do trabalho nos serviços de saúde. Em um contexto de lutas por melhores condições laborais e pela redução da carga horária é crucial que associações e sindicatos abordem essa questão.

## INTRODUÇÃO

A categoria da enfermagem demonstra, em relação a si própria, um interesse recente por aquilo que é um dos conteúdos da sua profissão – o tanto de carga de trabalho, excesso de obrigações, desgastes e riscos que são impostos aos trabalhadores. Ao contrário dos processos produtivos industriais, agrícolas ou comerciais, nos quais se transformam matérias-primas, o trabalho em saúde tem como objeto direto a vida humana. Nesse contexto, sua realização envolve dimensões que vão além das competências estritamente técnicas, incluindo a capacidade de lidar com o sofrimento das pessoas que são objeto do cuidado^(1)^.

Profissionais de saúde frequentemente encontram dificuldades para reconhecer, no próprio processo de trabalho, condições que possam contribuir para a ocorrência de agravos, adoecimentos e acidentes. Observa-se, muitas vezes, uma postura de forte comprometimento com o cuidado, na qual, mesmo diante de condições inadequadas, o trabalhador direciona sua energia à atenção ao indivíduo e à coletividade. Nesse movimento, tende a deixar em segundo plano a reflexão crítica e o questionamento de suas próprias condições de trabalho, sendo submetido a diferentes formas de sobrecarga física, emocional e cognitiva^(2)^. No entanto, quando se fala de cargas de trabalho ou de risco ocupacional, é preciso considerar, no limite, a produção de condições patológicas irreversíveis e incapacitantes, muitas das quais poderiam ser prevenidas. Além disso, a ação de saúde dirigida aos determinantes e condicionantes dos problemas de saúde ocupacional deveria visar, de modo ampliado, à promoção da saúde do trabalhador, numa perspectiva mais ampla de qualidade de vida e não apenas de prevenção pontual de problemas.

Vale discutir brevemente a distinção entre os conceitos de risco ocupacional e cargas de trabalho. O conceito de riscos ocupacionais vem sendo amplamente utilizado no marco teórico dos estudos epidemiológicos tradicionais, ancorado na perspectiva da saúde ocupacional e da higiene do trabalho, abordagens não implicadas em análises sobre a raiz das determinações históricas e políticas sobre a saúde dos trabalhadores.

Por sua vez, o conceito de cargas de trabalho, formulado e desenvolvido sobretudo no âmbito da Medicina Social latino-americana, parte de uma compreensão de saúde fundada na perspectiva da sua produção social, isto é, buscando compreender os fenômenos de saúde como históricos e produzidos sob dadas condições e relações sociais. A noção de riscos ocupacionais, apesar de ser operacional para identificar elementos isolados do trabalho capazes de aumentar a probabilidade de danos à saúde, é insuficiente porque não dá conta da complexidade do processo de desgaste, responsabilizando agentes específicos por doenças específicas e ignorando a patogênese mais ampla. É também ideológica porque, ao focar em agentes externos, desvia o foco da organização social do trabalho capitalista – que é a verdadeira raiz do desgaste – para soluções técnicas e paliativas que não contestam a lógica exploratória do capital. Portanto, enquanto “riscos ocupacionais” é uma categoria que individualiza, fragmenta e tecnifica o problema, “cargas de trabalho” é uma categoria que o socializa e politiza a questão, colocando no centro do debate a luta pela transformação das condições e da organização do trabalho^(3)^.

As mudanças devido ao processo de reestruturação produtiva promoveram modificações na natureza, no conteúdo e no significado do trabalho^(4)^. Nas últimas décadas, os processos de modernização tecnológica e de reorganização das estruturas produtivas têm produzido mudanças significativas na gestão do trabalho. Entre as principais características desse movimento destacam-se a intensificação do ritmo laboral, a ampliação da carga horária, a padronização e mecanização de tarefas, o controle mais rigoroso das atividades, a incorporação de metas aos processos de avaliação, a pressão exercida pelas chefias, a exigência de trabalhadores polivalentes e a precarização das relações de trabalho, entre outros aspectos^(4)^.

A partir da década de 1990, também surgem uma série de novas questões no trabalho em saúde, marcando um ponto de inflexão na análise e na pesquisa sobre o processo de trabalho em saúde. De um lado, emergiram preocupações relacionadas às novas modalidades de trabalho flexível ou informal, bem como à regulação promovida pelo Estado, que se concentrava nos mecanismos institucionais que gerenciam o trabalho^(2,5)^. De outro lado, há questões acerca da integralidade do cuidado e da autonomia dos indivíduos, cujo foco da análise se desloca para as interações envolvidas na relação entre profissionais e usuários, ou entre os próprios profissionais^(6)^.

A distinção entre o trabalho produtivo, como produtor de mais-valia, e o trabalho improdutivo, como realizador de mais-valia, que tem relevância sobretudo na análise do capital em si, não se apresenta com a mesma nitidez no plano das relações concretas. Como indicado por diferentes interpretações marxistas^(7)^, ser trabalhador produtivo ou improdutivo significa, imediatamente, ser explorado pelo capital. No âmbito estrito da produção capitalista^(8)^, a diferença não reside no que se produz — um bem material ou um serviço —, mas para quem e sob quais relações sociais o trabalho é realizado. No plano teórico (o “capital em si”), a distinção é clara e essencial para entender a gênese da mais-valia, que é o segredo da acumulação capitalista: o trabalho produtivo é a fonte da riqueza capitalista. Já no plano das relações concretas (a “aparência fenomênica”), esta distinção se esvai. O trabalho improdutivo é funcional e indispensável. A forma salarial unifica experiências díspares, e a complexificação do capitalismo (especialmente em países dependentes como o Brasil) cria um *continuum* de exploração, no qual a linha que separa produtivo de improdutivo é atravessada por relações de precariedade, subsunção real do trabalho ao capital financeiro e uma imensa massa de trabalhadores que, sem produzir mais-valia, são vitais para sua realização e para a reprodução da força de trabalho barata^(8,9)^.

A força de trabalho em enfermagem, assim como a de outras categorias que compõem a classe trabalhadora, encontra-se submetida às transformações recentes na gestão e organização do trabalho^(2)^. Nesse contexto, a fragmentação e a reorganização das atividades de enfermagem, frequentemente justificadas pela busca de maior eficiência e produtividade, tendem a tratar essa força de trabalho como uma mercadoria a ser mobilizada conforme as demandas do processo produtivo^(1)^.

O desafio, portanto, é o de identificar e analisar as mediações entre as dimensões macroestruturais do mundo do trabalho e da produção de riquezas, e as esferas cotidianas do trabalho em saúde, mediações essas que afetam e configuram determinados modos de organização do trabalho. Sob essa ótica, cabe também apontar, criticamente, para os desafios impostos às abordagens em saúde do trabalhador, em contrapartida aos limites dos campos da saúde ocupacional e da medicina do trabalho^(10)^. A literatura registra um número expressivo de estudos dedicados à saúde dos trabalhadores de enfermagem. Grande parte dessas investigações, entretanto, concentra-se na descrição de doenças e de riscos ocupacionais aos quais esses profissionais estão expostos^(11)^. Embora relevantes, tais abordagens tendem a oferecer uma compreensão parcial da realidade, uma vez que frequentemente deixam de examinar os determinantes das morbidades vinculadas ao processo de trabalho, restringindo-se a intervenções pontuais e não alcançando a análise das origens estruturais desses problemas.

A saúde dos profissionais de enfermagem é influenciada por diferentes dimensões da organização do trabalho, entre as quais se destacam a jornada laboral, a remuneração, o grau de autonomia, as condições de trabalho e as relações socioprofissionais^(12,13)^, fatores que também têm sido apontados como associados à saída de enfermeiros da profissão em países europeus^(14)^. Nesse sentido, com o emprego do conceito de intensificação do trabalho como objeto de análise, o estudo aprofunda questões identificadas em estudos anteriores, nos quais as mudanças e configurações da organização do trabalho de enfermagem se apresentavam já como importante campo de determinação de processos de saúde-doença do trabalhador dessa área, para além do enfoque pontual sobre riscos específicos a que ele estaria submetido.

Este estudo é uma produção acadêmica de um grupo de pesquisa certificado, voltado às relações entre trabalho, saúde dos trabalhadores e enfermagem, vinculado a um programa de pós-graduação na área da enfermagem, constituído por pesquisadores em diferentes níveis de formação. O presente trabalho tem como objetivos caracterizar e descrever, do ponto de vista teórico-conceitual, a organização do trabalho da enfermagem no modo de produção capitalista e discutir o fenômeno da intensificação do trabalho e suas implicações para o trabalho de enfermagem e a saúde do trabalhador.

## MÉTODO

Trata-se de um estudo teórico-reflexivo, sustentado na literatura científica contemporânea relativa ao tema da intensificação do trabalho na enfermagem e ancorado em uma perspectiva crítica de análise. Na primeira vertente, entre 2020 e 2025, foi realizada uma busca não sistemática de estudos que tratam diretamente do objeto de estudo. O filtro de escolha combinou “*intensificação do trabalho AND enfermagem*”, utilizando as bases de dados: Scientific Electronic Library Online – SciELO, Literatura Latino-Americana e do Caribe em Ciências da Saúde – Lilacs e Biblioteca Digital Brasileira de Teses e Dissertações (BDTB). Na base SciELO, foram selecionados quatro textos, sendo dois considerados pertinentes à temática. Na base Lilacs, nenhum artigo foi encontrado. E, na base BDTB, encontraram-se 31 teses ou dissertações, sendo selecionados dois que abordavam a temática de intensificação do trabalho como central. Após o filtro da redundância, foram selecionados três trabalhos. A revisão da literatura foi enriquecida por uma série de artigos que não abordavam diretamente o foco principal do estudo. No entanto, no decorrer da pesquisa, surgiram elementos relacionados à intensificação do trabalho em enfermagem.

Após o cotejamento da literatura disponível com o acúmulo das discussões desenvolvidas em grupos de estudo e em espaços formativos na área da enfermagem, o estudo foi organizado em dois eixos temáticos: dimensões teórico-conceituais do trabalho em enfermagem e sua organização no modo de produção capitalista; intensificação do trabalho e sua relação com a enfermagem e a saúde dos trabalhadores. Com base na teoria crítica do valor-trabalho, os principais pontos de discussão foram as características do trabalho em enfermagem e sua organização frente aos modos de acumulação em curso; e como se configura a intensificação do trabalho em enfermagem em vários cenários do setor de saúde, com suas consequências para o trabalho e a saúde dos trabalhadores.

## RESULTADOS

### Dimensões Teórico-Conceituais do Trabalho em Enfermagem e sua Organização no Modo de Produção Capitalista

A investigação sobre o trabalho da enfermagem ganhou enfoque a partir da década de 1980, sendo abordada sob diversas perspectivas teóricas. Do ponto de vista do trabalho como prática social, alguns autores consideram o trabalho de enfermagem como condicionado ao modelo de atenção de base biomédica hegemônico, ou seja, atrelado e subsumido ao trabalho médico^(15)^. Por outro lado, há também aqueles que analisam o trabalho em enfermagem como algo do âmbito do trabalho em saúde, em que diferentes profissionais colaboram de maneira a desenvolver processos específicos para compreender e intervir no processo de saúde e doença tanto dos indivíduos quanto da coletividade^(6)^.

Para conceituar o processo de trabalho em saúde, tomou-se como base as formulações teóricas que o compreendem a partir de quatro dimensões inter-relacionadas: a atividade em si (o fazer), o objeto de trabalho, os agentes (trabalhadores) e os instrumentos ou meios de trabalho^(6,16)^. A forma como esses elementos são definidos, se encadeiam e se articulam temporal e espacialmente expressa uma determinada forma de organização do trabalho. Com o desenvolvimento do capitalismo, a práxis da saúde passa a compor a relação entre processo de trabalho e de valorização, estando, assim, subordinada à continuidade do processo de acumulação capitalista e da reprodução das condições econômicas, políticas e ideológicas. Se, por um lado, o desenvolvimento dessa dinâmica resulta na formação de um complexo médico-industrial/financeiro que lucra com a compra e venda da mercadoria *saúde*; por outro, também se sustenta na exploração da força de trabalho responsável pela produção do cuidado em saúde^(17)^.

Com base nas análises clássicas de base crítica, em especial no que se tem denominado *teoria do valor-trabalho* em Marx, considera-se que a organização do trabalho, sob o modo capitalista, deixa de pertencer ao trabalhador e passa progressivamente às mãos dos que detêm os meios de produção^(8,18)^. Do ponto de vista da crítica marxiana, trabalho produtivo e improdutivo não se separam quando se considera o processo de acumulação de capital. Isso pode explicar, em parte, como diferentes formas de gestão, organização e divisão do trabalho, como o produtivo e o improdutivo, acabam por tomar como base os mesmos pressupostos teórico-políticos, científicos e pedagógicos.

É a subsunção do trabalho ao capital que leva à constituição de certas formas de gestão e administração do processo de trabalho. Nesse contexto, as formulações no âmbito da Administração Científica do Trabalho^(19)^ foram pioneiras e constituíram um conjunto de princípios que passaram a normatizar a vida operária, dentro e fora dos locais de trabalho, com o objetivo de aumento da exploração do trabalho e redução dos custos de produção, sendo essa lógica também utilizada, ao longo da história, para impulsionar outros modelos mais flexíveis de organização do trabalho, como o toyotista e o fluxo tensionado^(2)^.

Para a reflexão proposta, não é necessária uma exposição detalhada das peculiaridades dos diferentes sistemas de organização do trabalho, mas sim a compreensão de que, de alguma maneira, esses modos de organização influenciaram e ainda influenciam o trabalho coletivo em saúde e, especificamente, em enfermagem^(1,2)^. Nessa perspectiva, não há um modelo universal para o trabalho de enfermagem que possa ser aplicado a qualquer instituição ou unidade de saúde. Isso ocorre porque a variedade da atividade da enfermagem, que acompanha a diversificação na oferta de serviços, unidades e setores, implica a utilização de variados modelos visando à redução dos custos operacionais e ao aumento da eficiência dos profissionais. Ao mesmo tempo que a atividade da enfermagem revela características distintas, a gestão também se depara com formas de resistência, tanto individuais quanto coletivas, por parte dos trabalhadores que contestam as alterações implementadas em seus ambientes laborais^(5)^.

Nesse sentido, o trabalho de enfermagem compartilha, com as demais categorias de trabalhadores da saúde, da vivência de condições e mudanças nas formas de organização do trabalho que lhes são impostas pelos modelos de acumulação em curso. Identificam-se, de forma sintética, algumas dessas transformações, usualmente referidas como a precarização e flexibilização das condições de trabalho e emprego (com certa ênfase em questões de terceirização); mudanças na qualificação/formação profissional dos trabalhadores, com destaque para a primazia de uma *racionalidade pedagógica* instrumental, sobre a formativa; e implantação de programas derivados do chamado *modelo japonês*, como qualidade total, *just in time* etc., em especial no setor privado de saúde.

Já as transformações no setor público se tornam mais pronunciadas a partir dos anos 1990, quando é estabelecido um novo papel para o Estado. Nesse contexto, emerge o terceiro setor como uma resposta à alegação de que a administração pública seria incapaz de utilizar os recursos financeiros com eficiência. Isso leva a uma gradual substituição da burocracia hierárquica pública, na prestação de serviços à população, pelos princípios da gestão empresarial com foco na mensuração dos resultados, aumentando a autonomia gerencial e a eficiência e efetividade dos serviços, mediante desempenho e competição^(2,20)^. Assim, a evolução do capitalismo resultou em transformações importantes na organização do trabalho em enfermagem, aumentando a precarização do trabalho e exigindo um aumento do esforço físico, psicológico e intelectual dos trabalhadores. Nesse contexto, o fenômeno da intensificação do trabalho se tornou um aspecto central de análise nas transformações atuais dos processos produtivos^(4,21)^.

### Intensificação do Trabalho e sua Relação com a Enfermagem e a Saúde dos Trabalhadores

Apesar da crescente relevância do debate em torno da intensificação do trabalho, esse não é um fenômeno recente e já havia sido observado por Marx como presente nos primórdios do capitalismo^(4,21)^. A intensificação do trabalho pode ser apontada como o preenchimento mais denso dos poros do tempo de trabalho com o objetivo de comprimir um maior *quantum* de trabalho no mesmo intervalo de tempo, qual seja, o da jornada de trabalho. Esse fenômeno é descrito em estudos clássicos da organização científica do trabalho^(19)^, que descrevem que o aumento da produtividade aliou diminuição da jornada laboral e redução do efetivo entre as funcionárias responsáveis pela inspeção da qualidade de esferas de aço destinadas à fabricação de mancais de bicicletas. Anteriormente, 120 operárias cumpriam uma jornada de aproximadamente 10,5 horas diárias; após a análise do processo de trabalho, apenas 35 trabalhavam 8,5 horas por dia, conseguindo realizar suas tarefas com maior precisão, a uma velocidade 2/3 maior.

Dentre as alterações implementadas para aumentar a produtividade e reduzir a jornada destacam-se a seleção criteriosa de funcionárias mais qualificadas, a documentação diária da qualidade e do volume de trabalho realizado pelas operárias, o pagamento por peça, junto a uma gratificação adicional, e a adição de uma pausa de 10 minutos após 1,5 hora de trabalho. Há, portanto, evidente relação entre o conceito de intensificação e o de tempo de trabalho. Historicamente, a redução da jornada de trabalho tem sido associada à ideia de progresso social e à melhoria das condições de vida, constituindo-se como uma importante pauta das lutas coletivas dos trabalhadores em diferentes contextos nacionais^(22)^.

No Brasil, destacam-se dois marcos importantes relacionados à duração da jornada de trabalho: a regulamentação ocorrida na década de 1930, posteriormente incorporada à Consolidação das Leis do Trabalho, e os desdobramentos das greves dos metalúrgicos do ABC Paulista na década de 1980, que contribuíram para a redução da jornada semanal de 48 para 44 horas na Constituição Federal de 1988^(22)^. Ainda assim, após essas conquistas mais recentes, passaram a emergir novas formas de intensificação do trabalho. É importante notar que essa questão é especialmente relevante para a enfermagem no Brasil, atualmente, considerando a aprovação do piso salarial para a categoria (Lei nº 14.434, de 4 de agosto de 2022) e o trâmite, no Congresso Nacional, de várias outras propostas (Projeto de Lei nº 2.295/2000 e Proposta de Emenda à Constituição nº 19/2024) que visam estabelecer uma jornada máxima de 30 horas semanais para todos os profissionais de enfermagem.

Sem negar a importância da conquista da redução do tempo de trabalho, que certamente em muito contribuirá para a qualificação do trabalho da enfermagem, é preciso colocar em questão se, por outro lado, essa redução não poderá implicar em intensificação do trabalho, fenômeno percebido em outras categorias. Como mencionado anteriormente, na gestão dos serviços de saúde pressupostos econômicos e institucionais interagem, moldando e definindo a lógica de estruturação da organização de trabalho para acumular ou poupar capital^(20)^. Isso significa que, mesmo com diminuição da jornada, esses pressupostos se mantêm e podem dar origem a práticas (já conhecidas ou novas) que intensificam o trabalho dos profissionais de enfermagem.

A literatura tem apontado a intensificação do trabalho como uma dimensão particular do processo de exploração e expropriação do saber do trabalhador, e tem ocorrido por meio de uma combinação específica entre variados elementos do processo de trabalho, como o prolongamento do tempo de trabalho, o aumento do ritmo de trabalho, as exigências de polivalência, a gestão por desempenho/performance ou a administração por estresse^(21)^. A despeito de alguns estudos^(13,23,24)^, existe uma carência de investigações em que o tema da intensificação do trabalho em enfermagem e saúde aparece como objeto específico de investigação. Por outro lado, uma série de análises evidenciam o fenômeno da intensificação, ao destacar a presença de elementos que se aproximam ou estão implicados na dinâmica de trabalho de enfermagem nos serviços e unidades de saúde. A literatura descreve que o prolongamento do tempo de trabalho tem sido instituído pelo sistema de banco de horas, plantões extras e impossibilidade dos trabalhadores usufruírem, de fato, de um período de férias.

O sistema de banco de horas tem sido adotado em hospitais, tanto públicos quanto privados, em razão da falta de profissionais e das orientações de gestão^(25, 26)^, o que contribui para o aumento do estresse entre os trabalhadores de enfermagem, pois, devido às exigências institucionais, eles não conseguem usufruir das horas extras trabalhadas no momento que desejam fazê-lo^(26)^. Conforme destaca Felli^(25)^, é comum que os funcionários sejam levados pela administração a compensar horas extras durante os períodos de licença, devido a questões de saúde, mesmo quando teriam direito a afastamento por acidente ou atestado médico.

Os plantões extras, enquanto uma modalidade de prolongamento da jornada de trabalho, são uma prática recorrente na categoria. Essa dinâmica decorre tanto do número insuficiente de profissionais nas unidades e serviço de saúde quanto da baixa remuneração da força de trabalho, que não garante o sustento próprio e familiar. Esta prática foi descrita entre trabalhadores da categoria alocados em um hospital de ensino do estado do Paraná^(27)^ e em um serviço de tratamento oncológico em hospital especializado no estado do Rio de Janeiro, onde se registrou uma média de 50 horas semanais de trabalho, realizadas com o propósito de suprir o déficit de profissionais e complementar a renda diante aos baixos salários^(28)^.

O último aspecto da extensão da jornada de trabalho descrito em pesquisas é a ausência de aproveitamento das férias no período previsto. De acordo com informações de estudo sobre o perfil da enfermagem no Brasil, realizado em 2014, estima-se que aproximadamente 200 mil profissionais da área de enfermagem, representando em torno de 11% do total da força de trabalho, informaram não tirar férias anuais de maneira regular^(12)^. Desde então, esse número pode ter crescido ainda mais em razão do aumento da terceirização e de novas formas de contratação, por conta de alterações na legislação trabalhista.

Ao se reduzir o intervalo de descanso intra e interjornada, se diminui o tempo de recuperação da força de trabalho, pois os dias que deveriam ser utilizados para estudo, lazer ou, por direito, para compartilhar da vida social com familiares e amigos, são incorporados na jornada semanal ou mensal. Longas horas trabalhadas aumentam o desgaste físico e emocional e podem gerar uma série de agravos à saúde, como doenças crônicas não transmissíveis^(27)^ e diminuição do padrão de sono^(28)^.

O segundo elemento presente na intensificação do trabalho se encontra no aumento do ritmo de trabalho, que ocorre pela redução ou eliminação dos intervalos (*poros*) intra e interturnos, durante a jornada laboral. Dada a diversidade de serviços e unidades, o aumento do ritmo de trabalho da enfermagem contempla fatores como a redução do efetivo para a prestação da assistência necessária^(2,26)^, um quantitativo elevado de demandas a atender em um dado espaço de tempo^(2,5)^, a incorporação de tecnologia^(13)^ ou a constituição de metas para avaliação de desempenho^(5)^.

O elevado ritmo de trabalho foi descrito como um fator de intensificação do trabalho da enfermagem em unidades de clínica médica e cirúrgica do Hospital Universitário de Santa Maria^(23)^. Somam-se a esse processo a alta demanda de pacientes, o quantitativo reduzido de profissionais, o acúmulo de múltiplas tarefas ao longo da jornada e condições laborais inadequadas. Martins^(5)^, ao estudar o processo de reestruturação da atenção básica do Rio de Janeiro, descreve a intensificação do trabalho de enfermagem como resultado do aumento do número de atendimentos ao longo do dia, e do trabalho da enfermagem tratado como uma extensão do trabalho médico.

A incorporação de metas como forma de avaliação contínua dos trabalhadores é usada como ferramenta da gestão para intensificar o trabalho, em diferentes processos produtivos^(4,20)^, um elemento não considerado no instrumento para medir a intensificação do trabalho^(24)^. Entretanto, tal prática tem sido cada vez mais presente no trabalho em enfermagem. Um exemplo pode ser observado na relação entre o Estado e as organizações sociais (OS), na qual o contrato de gestão estabelece uma série de objetivos e metas^(20)^ que condicionam a organização do trabalho nas unidades de saúde. Nesse contexto, a finalidade imediata do processo produtivo dos serviços tende a assumir a forma de um produto quantificável, expresso no número de procedimentos, intervenções, consultas e outros atos de saúde realizados^(6)^. Nos processos de trabalho, as metas não são apenas um horizonte numérico, pois elas passam a ser definidoras da extensão e da intensidade da jornada de trabalho, bem como provocam a diminuição dos intervalos, por parte do próprio trabalhador. Além disso, podem condicionar salários efetivos e potenciais e gerar maior controle e competição entre os trabalhadores, ao mesmo tempo que repõem a heteronomia, produzindo um ambiente propício para a constituição de diversas formas de violência, de acordo com a hierarquia estabelecida^(4)^.

Essa racionalidade centrada em metas e resultados quantificáveis encontra ressonância em outra dimensão contemporânea do trabalho: a incorporação crescente das tecnologias digitais. A introdução de tecnologias digitais e automatizadas — vinculadas ao paradigma da chamada Indústria 4.0 — tem provocado profundas transformações na organização e na experiência laboral de trabalhadores dos mais diversos setores econômicos^(29,30)^.

Apesar da escassez de estudos sobre o tema no trabalho da enfermagem, é fundamental considerar as possibilidades do impacto e mediações produzidas por instrumentos como os sistemas de prontuário eletrônico, os softwares de gestão hospitalar e as novas formas de plataformização do trabalho em saúde, que podem reconfigurar tempos, modos e relações de trabalho. Tais inovações, embora legitimadas pelo discurso da eficiência e da modernização, podem estar contribuindo para intensificar os ritmos e ampliar o controle, reforçando a tendência de mensuração e vigilância que já marca o cotidiano do trabalho em enfermagem^(4)^, o que está descrito na plataformização do trabalho de diferentes trabalhadores do setor de serviço^(30)^ ou no trabalho médico^(31)^.

A plataformização, nesse sentido, expressa uma etapa avançada deste processo, na qual a mediação tecnológica reconfigura as formas de subordinação, os vínculos laborais e a própria dinâmica de prestação de serviços, introduzindo novas dimensões de precarização e desgaste no cotidiano dos profissionais^(30,31)^. Trata-se de um fenômeno claramente político de reorganização da exploração, na qual a intensificação do trabalho assume novas expressões — seja pelo deslocamento espacial e pelo controle mediado por ferramentas digitais de comunicação, seja pela convocação permanente destes trabalhadores por meio de dispositivos conectados^(30)^. Essa intensificação é escamoteada pela aparência de autonomia e pseudoliberdade do trabalhador, o que ganha contornos atraentes em contextos que visam estimular a figura do “empreendedor”. Na realidade, contudo, trata-se de um trabalhador explorado, exausto, precarizado, sem proteção social e submetido a jornadas indefinidas, cuja autonomia é apenas uma forma ideológica de mascarar a subordinação.

## CONSIDERAÇÕES FINAIS

Para concluir, depreende-se desta discussão que, por meio da análise do conceito de intensificação, é possível se tecer análises das relações entre níveis de determinação e condicionamento da saúde dos trabalhadores enfermeiros a fim de iluminar aspectos pouco visíveis, referidos a processos recentes de mudanças na organização do trabalho. Isso traz à tona um novo desafio: a necessidade de se adaptar a abordagem teórica e metodológica, assim como os métodos e ferramentas a tais mudanças, para avançar na análise da intensificação do trabalho de enfermagem como objeto de pesquisa. Para tal, é preciso não só compreender a dinâmica dos serviços de saúde, que estrutura e estabelece a forma de organização do trabalho, mas também as características próprias, a partir das experiências e vivências dos próprios trabalhadores, que tendem a encobrir formas de exploração do trabalho sustentadas por processos de subjetivação. Ressalte-se, no entanto, que dada a complexidade do fenômeno da intensificação e seus processos de determinação, é preciso indicar os limites do presente texto, que buscou abordar apenas alguns elementos mais visíveis e documentados na literatura, bem como debates recentes. Não foram explorados, portanto, categorias metodológicas e teóricas do pensamento marxiano, em especial as de mediação e totalidade, o que exigiria o desenvolvimento de um material mais longo e denso, incompatível com o formato de um artigo de reflexão.

Além de compreender os pressupostos presentes no serviços de saúde, ressaltam-se três aspectos para o estudo da intensificação do trabalho na enfermagem: primeiramente, a necessidade de compreender o trabalho de enfermagem no contexto do trabalho coletivo em saúde, uma vez que esses elementos podem se manifestar, de diversas maneiras, em diferentes categorias da área da saúde, afetando o coletivo de trabalhadores; e, em segundo lugar, a identificação das formas de resistência, tanto abertas quanto veladas, que confrontem o papel de gestão em relação às práticas de intensificação do trabalho usadas como mecanismo para enfraquecer a capacidade coletiva dos trabalhadores de defender a própria saúde. Em terceiro lugar, é fundamental entender que, além de um objeto acadêmico a ser desenvolvido, a intensificação do trabalho se materializa no cotidiano dos trabalhadores, o que provoca uma necessidade de debate nas associações e sindicatos da categoria.

Por fim, destaca-se que, diante de um cenário de luta por condições de trabalho mais adequadas e pela diminuição da jornada, é essencial abordar a intensificação do trabalho como uma questão que, de maneira tangível ou potencial, representa um risco à qualidade de vida e às condições laborais da enfermagem.

## Data Availability

Todo o conjunto de dados que dá suporte aos resultados deste estudo foi publicado no próprio artigo.

# Translation: La intensificación del trabajo: una cuestión teórico-práctica para la práctica de la enfermería

**DOI:** 10.1590/1980-220X-REEUSP-2025-0408es

**Authors:** Helena Maria Scherlowski Leal David, Hugo Pinto de Almeida

**Affiliations:** 1 Universidade do Estado do Rio de Janeiro, Faculdade de Enfermagem, Programa de Pós-Graduação em Enfermagem, Rio de Janeiro, RJ, Brasil.; 2 Universidade do Estado do Rio de Janeiro, Faculdade de Enfermagem, Rio de Janeiro, RJ, Brasil.

**Keywords:** Enfermería, Proceso de Trabajo en Salud, Carga de Trabajo, Condiciones de Trabajo Salud Laboral

## Abstract

**Objetivo:**

Analizar, desde una perspectiva teórico-práctica, el fenómeno de la intensificación del trabajo y sus implicaciones para la práctica de la enfermería y la salud de los trabajadores.

**Método:**

Se basa en la producción bibliográfica, mediante una búsqueda no sistemática sobre la intensificación del trabajo en enfermería, combinada con la experiencia y el pensamiento crítico de los autores.

**Resultados:**

A partir de la teoría crítica del valor-trabajo, se analizan las características del trabajo en enfermería y las influencias de las formas de organización del trabajo frente a los modos de acumulación vigentes. Se discute cómo se manifiesta la intensificación en diferentes escenarios del sector de la salud y sus repercusiones en el trabajo y en la salud de los trabajadores.

**Consideraciones finales:**

Aunque no se configura como un tema de investigación ampliamente consolidado, la intensificación del trabajo surge de distintas formas en la literatura académica. Para avanzar en la comprensión del objeto, resulta fundamental considerar las recientes transformaciones en la organización del trabajo en los servicios de salud. En un contexto de luchas por mejores condiciones laborales y por la reducción de la carga horaria, es crucial que las asociaciones y los sindicatos aborden esta cuestión.

## INTRODUCCIÓN

La profesión de enfermería muestra, en relación consigo misma, un interés reciente por lo que constituye uno de los aspectos de su profesión: la carga de trabajo, el exceso de obligaciones, el desgaste y los riesgos que se imponen a los trabajadores. A diferencia de los procesos productivos industriales, agrícolas o comerciales, en los que se transforman materias primas, el trabajo en salud tiene como objeto directo la vida humana. En este contexto, su realización implica dimensiones que van más allá de las competencias estrictamente técnicas, incluyendo la capacidad de lidiar con el sufrimiento de las personas que son objeto de los cuidados^(1)^.

Los profesionales de la salud suelen tener dificultades para reconocer, en el propio proceso de trabajo, las condiciones que pueden contribuir a la aparición de agravamientos, enfermedades y accidentes. A menudo se observa una postura de fuerte compromiso con el cuidado, en la que, incluso ante condiciones inadecuadas, el trabajador dirige su energía a la atención del individuo y de la colectividad. En este contexto, tiende a dejar en segundo plano la reflexión crítica y el cuestionamiento de sus propias condiciones de trabajo, quedando sometido a diferentes formas de sobrecarga física, emocional y cognitiva^(2)^. Sin embargo, cuando se habla de cargas de trabajo o de riesgo ocupacional, es necesario considerar, en última instancia, la aparición de condiciones patológicas irreversibles e incapacitantes, muchas de las cuales podrían prevenirse. Además, la acción de salud dirigida a los determinantes y condicionantes de los problemas de salud ocupacional debería apuntar, de manera más amplia, a la promoción de la salud del trabajador, desde una perspectiva más amplia de calidad de vida y no solo de prevención puntual de problemas.

Vale la pena discutir brevemente la distinción entre los conceptos de riesgo ocupacional y cargas de trabajo. El concepto de riesgos ocupacionales se ha utilizado ampliamente en el marco teórico de los estudios epidemiológicos tradicionales, anclado en la perspectiva de la salud ocupacional y la higiene del trabajo, enfoques que no implican análisis sobre la raíz de las determinaciones históricas y políticas sobre la salud de los trabajadores.

Por su parte, el concepto de cargas de trabajo, formulado y desarrollado sobre todo en el ámbito de la Medicina Social latinoamericana, parte de una comprensión de la salud basada en la perspectiva de su producción social, es decir, que busca entender los fenómenos de salud como históricos y producidos bajo determinadas condiciones y relaciones sociales. La noción de riesgos ocupacionales, a pesar de ser operativa para identificar elementos aislados del trabajo capaces de aumentar la probabilidad de daños a la salud, es insuficiente porque no da cuenta de la complejidad del proceso de desgaste, responsabilizando a agentes específicos por enfermedades específicas e ignorando la patogénesis más amplia. También es ideológica porque, al centrarse en agentes externos, desvía la atención de la organización social del trabajo capitalista —que es la verdadera raíz del desgaste— hacia soluciones técnicas y paliativas que no cuestionan la lógica explotadora del capital. Por lo tanto, mientras que “riesgos ocupacionales” es una categoría que individualiza, fragmenta y tecnifica el problema, “cargas de trabajo” es una categoría que socializa y politiza la cuestión, colocando en el centro del debate la lucha por la transformación de las condiciones y la organización del trabajo^(3)^.

Los cambios debidos al proceso de reestructuración productiva han promovido modificaciones en la naturaleza, el contenido y el significado del trabajo^(4)^. En las últimas décadas, los procesos de modernización tecnológica y de reorganización de las estructuras productivas han producido cambios significativos en la gestión del trabajo. Entre las principales características de este movimiento se destacan la intensificación del ritmo laboral, la ampliación de la jornada laboral, la estandarización y mecanización de las tareas, el control más riguroso de las actividades, la incorporación de metas a los procesos de evaluación, la presión ejercida por los superiores, la exigencia de trabajadores polivalentes y la precarización de las relaciones laborales, entre otros aspectos^(4)^.

A partir de la década de 1990, también surgen una serie de nuevas cuestiones en el trabajo en el ámbito de la salud, lo que marca un punto de inflexión en el análisis y la investigación sobre el proceso de trabajo en este sector. Por un lado, surgieron preocupaciones relacionadas con las nuevas modalidades de trabajo flexible o informal, así como con la regulación promovida por el Estado, que se concentraba en los mecanismos institucionales que gestionan el trabajo^(2,5)^. Por otro lado, hay cuestiones sobre la integralidad de la atención y la autonomía de los individuos, cuyo foco de análisis se desplaza hacia las interacciones involucradas en la relación entre profesionales y usuarios, o entre los propios profesionales^(6)^.

La distinción entre el trabajo productivo, como generador de plusvalía, y el trabajo improductivo, como realizador de plusvalía, que tiene relevancia sobre todo en el análisis del capital en sí, no se presenta con la misma claridad en el plano de las relaciones concretas. Como indican diferentes interpretaciones marxistas^(7)^, ser trabajador productivo o improductivo significa, de manera inmediata, ser explotado por el capital. En el ámbito estricto de la producción capitalista^(8)^, la diferencia no reside en lo que se produce —un bien material o un servicio—, sino para quién y bajo qué relaciones sociales se realiza el trabajo.

En el plano teórico (el “capital en sí”), la distinción es clara y esencial para comprender la génesis de la plusvalía, que es el secreto de la acumulación capitalista: el trabajo productivo es la fuente de la riqueza capitalista. Sin embargo, en el plano de las relaciones concretas (la “apariencia fenoménica”), esta distinción se desvanece. El trabajo improductivo es funcional e indispensable. La forma salarial unifica experiencias dispares, y la complejización del capitalismo (especialmente en países dependientes como Brasil) crea un *continuo* de explotación, en el que la línea que separa lo productivo de lo improductivo se ve atravesada por relaciones de precariedad, la subsunción real del trabajo al capital financiero y una inmensa masa de trabajadores que, sin producir plusvalía, es vital para su realización y para la reproducción de la fuerza de trabajo barata^(8,9)^.

La fuerza de trabajo en enfermería, al igual que la de otras categorías que componen la clase trabajadora, se encuentra sometida a las recientes transformaciones en la gestión y organización del trabajo^(2)^. En este contexto, la fragmentación y la reorganización de las actividades de enfermería, a menudo justificadas por la búsqueda de una mayor eficiencia y productividad, tienden a tratar a esta fuerza de trabajo como una mercancía que debe movilizarse según las demandas del proceso productivo^(1)^.

El desafío, por lo tanto, es identificar y analizar las mediaciones entre las dimensiones macroestructurales del mundo del trabajo y de la producción de riqueza, y las esferas cotidianas del trabajo en salud, mediaciones que afectan y configuran determinados modos de organización del trabajo. Desde esta perspectiva, también cabe señalar, de manera crítica, los desafíos que se plantean a los enfoques de la salud del trabajador, frente a los límites de los campos de la salud ocupacional y la medicina del trabajo^(10)^. La literatura registra un número significativo de estudios dedicados a la salud de los trabajadores de enfermería. Gran parte de estas investigaciones, sin embargo, se concentra en la descripción de enfermedades y riesgos ocupacionales a los que están expuestos estos profesionales^(11)^. Aunque relevantes, estos enfoques tienden a ofrecer una comprensión parcial de la realidad, ya que con frecuencia dejan de examinar los determinantes de las morbilidades vinculadas al proceso de trabajo, limitándose a intervenciones puntuales y sin llegar a analizar los orígenes estructurales de estos problemas.

La salud de los profesionales de enfermería se ve influida por diferentes dimensiones de la organización del trabajo, entre las que destacan la jornada laboral, la remuneración, el grado de autonomía, las condiciones de trabajo y las relaciones socioprofesionales^(12,13)^, factores que también se han señalado como asociados a la salida de enfermeros de la profesión en países europeos^(14)^. En este sentido, al emplear el concepto de intensificación del trabajo como objeto de análisis, el estudio profundiza en cuestiones identificadas en estudios anteriores, en los que los cambios y las configuraciones de la organización del trabajo de enfermería ya se presentaban como un campo importante para determinar los procesos de salud-enfermedad del trabajador de esta área, más allá del enfoque puntual sobre los riesgos específicos a los que estaría sometido.

Este estudio es una producción académica de un grupo de investigación certificado, enfocado en las relaciones entre el trabajo, la salud de los trabajadores y la enfermería, vinculado a un programa de posgrado en el área de la enfermería, constituido por investigadores en diferentes niveles de formación. El presente trabajo tiene como objetivos caracterizar y describir, desde un punto de vista teórico-conceptual, la organización del trabajo de enfermería en el modo de producción capitalista y discutir el fenómeno de la intensificación del trabajo y sus implicaciones para el trabajo de enfermería y la salud del trabajador.

## MÉTODO

Se trata de un estudio teórico-reflexivo, sustentado en la literatura científica contemporánea relativa al tema de la intensificación del trabajo en enfermería y anclado en una perspectiva crítica de análisis. En la primera fase, entre 2020 y 2025, se realizó una búsqueda no sistemática de estudios que trataran directamente el objeto de estudio. El filtro de selección combinó “*intensificação do trabalho AND enfermagem*”, utilizando las bases de datos: Scientific Electronic Library Online – SciELO, Literatura Latinoamericana y del Caribe en Ciencias de la Salud – Lilacs y Biblioteca Digital Brasileña de Tesis y Disertaciones (BDTB). En la base SciELO, se seleccionaron cuatro textos, dos de los cuales se consideraron pertinentes para la temática. En la base Lilacs no se encontró ningún artículo. Además, en la base BDTB, se encontraron 31 tesis o disertaciones, de las cuales se seleccionaron dos que abordaban el tema de la intensificación del trabajo como tema central. Tras el filtro de redundancia, se seleccionaron tres trabajos. La revisión de la literatura se enriqueció con una serie de artículos que no abordaban directamente el enfoque principal del estudio. Sin embargo, en el transcurso de la investigación, surgieron elementos relacionados con la intensificación del trabajo en enfermería.

Tras la comparación de la literatura disponible con el conjunto de los debates desarrollados en grupos de estudio y en espacios formativos en el área de la enfermería, el estudio se organizó en dos ejes temáticos: dimensiones teórico-conceptuales del trabajo en enfermería y su organización en el modo de producción capitalista; intensificación del trabajo y su relación con la enfermería y la salud de los trabajadores. Basándose en la teoría crítica del valor-trabajo, los principales puntos de discusión fueron las características del trabajo de enfermería y su organización frente a los modos de acumulación en curso; y cómo se configura la intensificación del trabajo de enfermería en diversos escenarios del sector de la salud, con sus consecuencias para el trabajo y la salud de los trabajadores.

## RESULTADOS

### Dimensiones Teórico-Conceptuales del Trabajo de Enfermería y su Organización en el Modo de Producción Capitalista

La investigación sobre el trabajo de enfermería cobró importancia a partir de la década de 1980, abordándose desde diversas perspectivas teóricas. Desde el punto de vista del trabajo como práctica social, algunos autores consideran que el trabajo de enfermería está condicionado al modelo de atención de base biomédica hegemónico, es decir, vinculado y subsumido al trabajo médico^(15)^. Por otro lado, también hay quienes analizan el trabajo de enfermería como algo que pertenece al ámbito del trabajo en salud, en el que diferentes profesionales colaboran para desarrollar procesos específicos destinados a comprender e intervenir en el proceso de salud y enfermedad tanto de los individuos como de la colectividad^(6)^.

Para conceptualizar el proceso de trabajo en salud, se tomaron como base las formulaciones teóricas que lo comprenden a partir de cuatro dimensiones interrelacionadas: la actividad en sí (el hacer), el objeto de trabajo, los agentes (trabajadores) y los instrumentos o medios de trabajo^(6,16)^. La forma en que estos elementos se definen, encadenan y articulan temporal y espacialmente expresa una determinada forma de organización del trabajo. Con el desarrollo del capitalismo, la praxis de la salud pasa a componer la relación entre el proceso de trabajo y de valorización, quedando así subordinada a la continuidad del proceso de acumulación capitalista y de la reproducción de las condiciones económicas, políticas e ideológicas. Si, por un lado, el desarrollo de esta dinámica da lugar a la formación de un complejo médico-industrial/financiero que se lucra con la compra y venta de la mercancía *salud*; por otro, también se sustenta en la explotación de la fuerza de trabajo responsable de la producción de la atención de salud^(17)^.

Basándose en los análisis clásicos de carácter crítico, en especial en lo que se ha denominado *teoría del valor-trabajo* en Marx, se considera que la organización del trabajo, bajo el modo capitalista, deja de pertenecer al trabajador y pasa progresivamente a manos de quienes poseen los medios de producción^(8,18)^. Desde el punto de vista de la crítica marxista, el trabajo productivo y el improductivo no se separan cuando se considera el proceso de acumulación de capital. Esto puede explicar, en parte, cómo diferentes formas de gestión, organización y división del trabajo, como el productivo y el improductivo, terminan basándose en los mismos supuestos teórico-políticos, científicos y pedagógicos.

Es la subsunción del trabajo al capital lo que conduce a la constitución de ciertas formas de gestión y administración del proceso de trabajo. En este contexto, las formulaciones en el ámbito de la Administración Científica del Trabajo^(19)^ fueron pioneras y constituyeron un conjunto de principios que pasaron a normatizar la vida obrera, tanto dentro como fuera de los lugares de trabajo, con el objetivo de aumentar la explotación del trabajo y reducir los costos de producción, siendo esta lógica también utilizada, a lo largo de la historia, para impulsar otros modelos más flexibles de organización del trabajo, como el toyotismo y el flujo tensionado^(2)^.

Para la reflexión propuesta, no es necesaria una exposición detallada de las peculiaridades de los diferentes sistemas de organización del trabajo, sino más bien la comprensión de que, de alguna manera, estos modos de organización influyeron y aún influyen en el trabajo colectivo en salud y, específicamente, en enfermería^(1,2)^. Desde esta perspectiva, no existe un modelo universal para el trabajo de enfermería que pueda aplicarse a cualquier institución o unidad de salud. Esto se debe a que la variedad de la actividad de enfermería, que acompaña a la diversificación en la oferta de servicios, unidades y sectores, implica la utilización de diversos modelos con el objetivo de reducir los costos operativos y aumentar la eficiencia de los profesionales. Al mismo tiempo que la actividad de enfermería revela características distintas, la gestión también se enfrenta a formas de resistencia, tanto individuales como colectivas, por parte de los trabajadores que cuestionan los cambios implementados en sus entornos laborales^(5)^.

En este sentido, el trabajo de enfermería comparte, con el resto de categorías de trabajadores de la salud, la experiencia de condiciones y cambios en las formas de organización del trabajo que les imponen los modelos de acumulación vigentes. Se identifican, de manera sintética, algunas de estas transformaciones, a las que se suele referir como la precarización y flexibilización de las condiciones de trabajo y empleo (con cierto énfasis en cuestiones de subcontratación); cambios en la cualificación/formación profesional de los trabajadores, destacando la primacía de una *racionalidad pedagógica* instrumental sobre la formativa; y la implantación de programas derivados del llamado *modelo japonés*, como la calidad total, *just in time*, etc., especialmente en el sector privado de la salud.

Por su parte, las transformaciones en el sector público se vuelven más pronunciadas a partir de la década de 1990, cuando se establece un nuevo papel para el Estado. En este contexto, surge el tercer sector como respuesta a la alegación de que la administración pública sería incapaz de utilizar los recursos financieros de manera eficiente. Esto lleva a una sustitución gradual de la burocracia pública jerárquica, en la prestación de servicios a la población, por los principios de la gestión empresarial centrados en la medición de resultados, aumentando la autonomía gerencial y la eficiencia y eficacia de los servicios, mediante el desempeño y la competencia^(2,20)^. Así, la evolución del capitalismo ha dado lugar a importantes transformaciones en la organización del trabajo en enfermería, aumentando la precariedad laboral y exigiendo un mayor esfuerzo físico, psicológico e intelectual de los trabajadores. En este contexto, el fenómeno de la intensificación del trabajo se ha convertido en un aspecto central de análisis en las transformaciones actuales de los procesos productivos^(4,21)^.

### La Intensificación del Trabajo y su Relación con la Enfermería y la Salud de los Trabajadores

A pesar de la creciente relevancia del debate en torno a la intensificación del trabajo, este no es un fenómeno reciente y ya había sido observado por Marx como presente en los primeros tiempos del capitalismo^(4,21)^. La intensificación del trabajo puede definirse como el relleno más denso de los poros del tiempo de trabajo con el objetivo de comprimir un mayor *quantum* de trabajo en el mismo intervalo de tiempo, es decir, el de la jornada laboral. Este fenómeno se describe en estudios clásicos sobre la organización científica del trabajo^(19)^, que señalan que el aumento de la productividad se acompañó de una disminución de la jornada laboral y una reducción de la plantilla entre las empleadas encargadas de la inspección de calidad de las esferas de acero destinadas a la fabricación de rodamientos de bicicletas. Anteriormente, 120 obreras cumplían una jornada de aproximadamente 10,5 horas diarias; tras el análisis del proceso de trabajo, solo 35 trabajaban 8,5 horas al día, logrando realizar sus tareas con mayor precisión, a una velocidad 2/3 mayor.

Entre los cambios implementados para aumentar la productividad y reducir la jornada laboral, se destacan la selección cuidadosa de las trabajadoras más calificadas, la documentación diaria de la calidad y el volumen de trabajo realizado por las obreras, el pago por pieza, junto con una gratificación adicional, y la incorporación de un descanso de 10 minutos después de 1,5 horas de trabajo.

Existe, por lo tanto, una relación evidente entre el concepto de intensificación y el de tiempo de trabajo. Históricamente, la reducción de la jornada laboral se ha asociado a la idea de progreso social y a la mejora de las condiciones de vida, constituyéndose como un tema importante de las luchas colectivas de los trabajadores en diferentes contextos nacionales^(22)^.

En Brasil, destacan dos hitos importantes relacionados con la duración de la jornada laboral: la reglamentación que tuvo lugar en la década de 1930, posteriormente incorporada a la Consolidación de las Leyes del Trabajo, y las repercusiones de las huelgas de los metalúrgicos del ABC Paulista en la década de 1980, que contribuyeron a la reducción de la jornada semanal de 48 a 44 horas en la Constitución Federal de 1988^(22)^. Aun así, tras estos logros más recientes, comenzaron a surgir nuevas formas de intensificación del trabajo. Es importante señalar que esta cuestión es especialmente relevante para la enfermería en Brasil en la actualidad, considerando la aprobación del salario mínimo para la categoría (Ley n.º 14.434, del 4 de agosto de 2022) y la tramitación, en el Congreso Nacional, de varias otras propuestas (Proyecto de Ley n.º 2.295/2000 y Propuesta de Enmienda a la Constitución n.º 19/2024) que tienen como objetivo establecer una jornada máxima de 30 horas semanales para todos los profesionales de enfermería.

Sin negar la importancia del logro que supone la reducción de la jornada laboral, que sin duda contribuirá en gran medida a la cualificación del trabajo de enfermería, es necesario cuestionar si, por otro lado, dicha reducción no podría implicar una intensificación del trabajo, fenómeno observado en otras categorías. Como se mencionó anteriormente, en la gestión de los servicios de salud interactúan supuestos económicos e institucionales, moldeando y definiendo la lógica de estructuración de la organización del trabajo para acumular o ahorrar capital^(20)^. Esto significa que, incluso con la reducción de la jornada, estos supuestos se mantienen y pueden dar lugar a prácticas (ya conocidas o nuevas) que intensifican el trabajo de los profesionales de enfermería.

La literatura ha señalado la intensificación del trabajo como una dimensión particular del proceso de explotación y expropiación del saber del trabajador, y ha ocurrido mediante una combinación específica entre diversos elementos del proceso de trabajo, como la prolongación del tiempo de trabajo, el aumento del ritmo de trabajo, las exigencias de polivalencia, la gestión por desempeño o el manejo del estrés^(21)^. A pesar de algunos estudios^(13,23,24)^, existe una carencia de investigaciones en las que el tema de la intensificación del trabajo en enfermería y salud aparezca como objeto específico de investigación. Por otro lado, una serie de análisis evidencian el fenómeno de la intensificación, al destacar la presencia de elementos que se acercan o están implicados en la dinámica del trabajo de enfermería en los servicios y unidades de salud. La literatura describe que la prolongación del tiempo de trabajo se ha instituido mediante el sistema de banco de horas, turnos extras y la imposibilidad de que los trabajadores disfruten, de hecho, de un período de vacaciones.

El sistema de banco de horas se ha adoptado en hospitales, tanto públicos como privados, debido a la falta de profesionales y a las orientaciones de la gestión^(25,26)^, lo que contribuye al aumento del estrés entre los trabajadores de enfermería, ya que, debido a las exigencias institucionales, no pueden disfrutar de las horas extras trabajadas en el momento en que desean hacerlo^(26)^. Como destaca Felli^(25)^, es común que la administración obligue a los empleados a compensar las horas extras durante los períodos de licencia por motivos de salud, incluso cuando tendrían derecho a una baja por accidente o certificado médico.

Los turnos extra, como forma de prolongar la jornada laboral, son una práctica recurrente en la categoría. Esta dinámica se debe tanto al número insuficiente de profesionales en las unidades y servicios de salud como a la baja remuneración de la fuerza laboral, que no garantiza el sustento propio ni el de la familia. Esta práctica se describió entre trabajadores del sector asignados a un hospital universitario del estado de Paraná^(27)^ y a un servicio de tratamiento oncológico en un hospital especializado del estado de Río de Janeiro, donde se registró un promedio de 50 horas semanales de trabajo, realizadas con el propósito de suplir el déficit de profesionales y complementar los ingresos ante los bajos salarios^(28)^.

El último aspecto de la prolongación de la jornada laboral descrito en las investigaciones es la falta de posibilidad de gozar las vacaciones en el período previsto. Según datos de un estudio sobre el perfil de la enfermería en Brasil, realizado en 2014, se estima que aproximadamente 200 mil profesionales del área de enfermería, lo que representa alrededor del 11% del total de la fuerza laboral, informaron no tomar vacaciones anuales de manera regular^(12)^. Desde entonces, esta cifra puede haber aumentado aún más debido al incremento de la subcontratación y a las nuevas formas de contratación, a raíz de los cambios en la legislación laboral.

Al reducirse los intervalos de descanso intra e interjornada, se disminuye el tiempo de recuperación de la fuerza laboral, ya que los días que deberían utilizarse para el estudio, el ocio o, por derecho, para compartir la vida social con familiares y amigos, se incorporan a la jornada semanal o mensual. Las largas jornadas laborales aumentan el desgaste físico y emocional y pueden generar una serie de problemas de salud, como enfermedades crónicas no transmisibles^(27)^ y la alteración de los patrones de sueño^(28)^.

El segundo elemento presente en la intensificación del trabajo se encuentra en el aumento del ritmo de trabajo, que se produce por la reducción o eliminación de los intervalos (*poros*) intra e interturnos, durante la jornada laboral. Dada la diversidad de servicios y unidades, el aumento del ritmo de trabajo de enfermería contempla factores como la reducción del personal para la prestación de la asistencia necesaria^(2,26)^, un elevado número de demandas que atender en un determinado espacio de tiempo^(2,5)^, la incorporación de tecnología^(13)^ o el establecimiento de metas para la evaluación del desempeño^(5)^.

El elevado ritmo de trabajo se describió como un factor de intensificación del trabajo de enfermería en las unidades de medicina clínica y quirúrgica del Hospital Universitario de Santa María^(23)^. A este proceso se suman la alta demanda de pacientes, el número reducido de profesionales, la acumulación de múltiples tareas a lo largo de la jornada y las condiciones laborales inadecuadas. Martins^(5)^, al estudiar el proceso de reestructuración de la atención primaria en Río de Janeiro, describe la intensificación del trabajo de enfermería como resultado del aumento del número de atendimientos a lo largo del día, y del trabajo de enfermería tratado como una extensión del trabajo médico.

La incorporación de metas como forma de evaluación continua de los trabajadores se utiliza como herramienta de gestión para intensificar el trabajo en diferentes procesos productivos^(4,20)^, un elemento no considerado en el instrumento para medir la intensificación del trabajo^(24)^. Sin embargo, dicha práctica ha estado cada vez más presente en el trabajo de enfermería. Se puede observar un ejemplo en la relación entre el Estado y las organizaciones sociales (OS), en la que el contrato de gestión establece una serie de objetivos y metas^(20)^ que condicionan la organización del trabajo en las unidades de salud. En este contexto, la finalidad inmediata del proceso productivo de los servicios tiende a adoptar la forma de un producto cuantificable, expresado en el número de procedimientos, intervenciones, consultas y otros actos de salud realizados^(6)^. En los procesos de trabajo, las metas no son solo un horizonte numérico, ya que pasan a definir la extensión y la intensidad de la jornada laboral, además de provocar la reducción de los descansos por parte del propio trabajador. Además, pueden condicionar los salarios efectivos y potenciales y generar un mayor control y competencia entre los trabajadores, al tiempo que restablecen la heteronomía, produciendo un ambiente propicio para la constitución de diversas formas de violencia, de acuerdo con la jerarquía establecida^(4)^.

Esta racionalidad centrada en metas y resultados cuantificables encuentra resonancia en otra dimensión contemporánea del trabajo: la creciente incorporación de las tecnologías digitales. La introducción de tecnologías digitales y automatizadas —vinculadas al paradigma de la llamada Industria 4.0— ha provocado profundas transformaciones en la organización y en la experiencia laboral de los trabajadores de los más diversos sectores económicos^(29,30)^.

A pesar de la escasez de estudios sobre el tema en el ámbito de la enfermería, es fundamental considerar las posibilidades del impacto y las mediaciones producidas por instrumentos como los sistemas de historias clínicas electrónicas, los programas de gestión hospitalaria y las nuevas formas de “plataformización” del trabajo en salud, que pueden reconfigurar los tiempos, los modos y las relaciones de trabajo. Tales innovaciones, aunque legitimadas por el discurso de la eficiencia y la modernización, pueden estar contribuyendo a intensificar los ritmos y ampliar el control, reforzando la tendencia a la medición y la vigilancia que ya caracteriza el día a día del trabajo de enfermería^(4)^, lo cual se describe en la platformización del trabajo de diferentes trabajadores del sector de servicios^(30)^ o en el trabajo médico^(31)^.

La “plataformización”, en este sentido, expresa una etapa avanzada de este proceso, en la que la mediación tecnológica reconfigura las formas de subordinación, los vínculos laborales y la propia dinámica de la prestación de servicios, introduciendo nuevas dimensiones de precariedad y desgaste en el día a día de los profesionales^(30,31)^. Se trata de un fenómeno claramente político de reorganización de la explotación, en el que la intensificación del trabajo adopta nuevas expresiones —ya sea por el desplazamiento espacial y el control mediado por herramientas digitales de comunicación, ya sea por la convocatoria permanente de estos trabajadores a través de dispositivos conectados^(30)^. Esta intensificación se disimula tras la apariencia de autonomía y pseudo-libertad del trabajador, lo que adquiere contornos atractivos en contextos que buscan estimular la figura del “emprendedor”. En realidad, sin embargo, se trata de un trabajador explotado, exhausto, precarizado, sin protección social y sometido a jornadas indefinidas, cuya autonomía no es más que una forma ideológica de enmascarar la subordinación.

## CONSIDERACIONES FINALES

Para concluir, de este debate se desprende que, mediante el análisis del concepto de intensificación, es posible realizar análisis de las relaciones entre los niveles de determinación y condicionamiento de la salud de los trabajadores de enfermería con el fin de esclarecer aspectos poco visibles, relacionados con procesos recientes de cambios en la organización del trabajo. Esto pone de manifiesto un nuevo desafío: la necesidad de adaptar el enfoque teórico y metodológico, así como los métodos y herramientas a dichos cambios, para avanzar en el análisis de la intensificación del trabajo de enfermería como objeto de investigación. Para ello, es necesario no solo comprender la dinámica de los servicios de salud, que estructura y establece la forma de organización del trabajo, sino también las características propias, a partir de las experiencias y vivencias de los propios trabajadores, que tienden a encubrir formas de explotación laboral sustentadas por procesos de subjetivación. Cabe destacar, sin embargo, que dada la complejidad del fenómeno de la intensificación y sus procesos de determinación, es necesario señalar los límites del presente texto, que ha buscado abordar solo algunos elementos más visibles y documentados en la literatura, así como debates recientes. Por lo tanto, no se han explorado las categorías metodológicas y teóricas del pensamiento marxista, en especial las de mediación y totalidad, lo que exigiría el desarrollo de un material más extenso y denso, incompatible con el formato de un artículo de reflexión.

Además de comprender los supuestos presentes en los servicios de salud, se destacan tres aspectos para el estudio de la intensificación del trabajo en enfermería: en primer lugar, la necesidad de comprender el trabajo de enfermería en el contexto del trabajo colectivo en salud, ya que estos elementos pueden manifestarse, de diversas maneras, en diferentes categorías del área de la salud, afectando al colectivo de trabajadores; y, en segundo lugar, la identificación de las formas de resistencia, tanto abiertas como veladas, que se oponen al papel de la gestión en relación con las prácticas de intensificación del trabajo utilizadas como mecanismo para debilitar la capacidad colectiva de los trabajadores de defender su propia salud. En tercer lugar, es fundamental comprender que, más allá de ser un tema académico a desarrollar, la intensificación del trabajo se materializa en la vida cotidiana de los trabajadores, lo que genera la necesidad de un debate en las asociaciones y sindicatos del sector.

Por último, cabe destacar que, ante un escenario de lucha por condiciones laborales más adecuadas y por la reducción de la jornada, es esencial abordar la intensificación del trabajo como una cuestión que, de manera tangible o potencial, representa un riesgo para la calidad de vida y las condiciones laborales de la enfermería.

## Data Availability

Todo el conjunto de datos que apoya los resultados de este estudio fue publicado en el propio artículo.
